# The inhibitory receptor LILRB4 (ILT3) modulates antigen presenting cell phenotype and, along with LILRB2 (ILT4), is upregulated in response to *Salmonella *infection

**DOI:** 10.1186/1471-2172-10-56

**Published:** 2009-10-27

**Authors:** Damien P Brown, Des C Jones, Katie J Anderson, Nicolas Lapaque, Robin A Buerki, John Trowsdale, Rachel L Allen

**Affiliations:** 1 Centre for Infection, Division of Cellular and Molecular Medicine, St Georges, University of London, London, UK; 2 Department of Pathology, Cambridge University, Cambridge, UK; 3 NorthWestern University, Evanston, USA

## Abstract

**Background:**

Leukocyte Ig-like receptors (LILR) are a family of innate immune receptors with immunomodulatory functions. High-level expression of the receptors LILRB2 (ILT4) and LILRB4 (ILT3) is a feature of tolerogenic antigen presenting cells and has been observed in cancer and transplant situations. There are relatively few studies regarding these receptors in the context of infection and it is not yet clear how LILRB4 exerts its inhibitory effects.

**Results:**

We studied the effects of LILRB4 ligation on antigen presenting cell phenotype, and the expression of LILRB2 and LILRB4 on *Salmonella-*infected antigen presenting cells. Ligation of LILRB4 throughout *in vitro *culture of dendritic cells led to an upregulation of the co-stimulatory protein CD86. Alterations in the production of IL-8 and IL-10 by LILRB4-ligated macrophages were also observed. Infection with *Salmonella typhimurium *or TLR stimulation with *Salmonella *components led to an upregulation of *LILRB2 *and *LILRB4*.

**Conclusion:**

Our results indicate that the inhibitory effects of LILRB4 do not result from a failure to upregulate co-stimulatory proteins. In addition to the high level expression that can render antigen presenting cells tolerogenic, there may be a role for lower level expression and activity of LILRB2 and LILRB4 in response to TLR signalling during an immune response to bacterial infection.

## Background

Leukocyte Ig-like receptors (LILR, also known as ILT and LIR) are a family of innate immune receptors that are predominantly expressed on cells of the myelomonocytic lineage [1]. The 11 members of the LILR protein family are divided into 'activating' (subfamily A) or 'inhibitory' (subfamily B) receptors on the basis of their transmembrane and cytoplasmic domains. There is a growing interest in LILR expression and/or function in situations of immune dysregulation and following transplantation [2-4], but as innate immune receptors they would also be predicted to play a role in an immune response to infection.

LILRB4 was first identified as an inhibitory receptor expressed on myeloid antigen presenting cells which associates with the protein tyrosine phosphotase SHP-1 [5]. The importance of this particular receptor as an immune modulator was highlighted when it was shown that high level expression of LILRB4 with LILRB2 rendered antigen presenting cells tolerogenic [4,6,7]. LILRB4 and LILRB2 may differ in their mechanisms of immune regulation. In receptor ligation studies, the tolerogenic properties of LILRB2 appear to result from phenotypic changes to the antigen presenting cells that express them, including failure to upregulate co-stimulatory proteins and MHC class II [8,9]. In contrast, although no receptor ligation studies have been performed for LILRB4 on antigen presenting cells, at least some of its inhibitory effects may result from reverse signalling through its T cell ligand, as soluble forms of LILRB4 have been shown to suppress T cell responses [10,11]. LILRB4 ligation was therefore performed to determine the phenotypic effects of receptor engagement on antigen presenting cell phenotype.

Given their potential to inhibit T cell proliferation, LILRB2 and LILRB4 have been studied in the context of allograft tolerance during transplantation and during cancer [4,10,12,13]. The ability of LILRB2 and LILRB4 to modulate T cell responses could also be of relevance during infection, where skewing of LILRB4 and/or LILRB2 expression might alter the course of an immune response. To better understand the inhibitory potential of LILRB2 and LILRB4 during pathogenic challenge, we set out to determine how their expression is modulated on antigen presenting cells infected with *Salmonella typhimurium*, as this obligate intracellular pathogen infects macrophages.

## Results

### Cell surface phenotype and T cell stimulatory capacity of LILRB4-ligated dendritic cells

**Figure 1:**
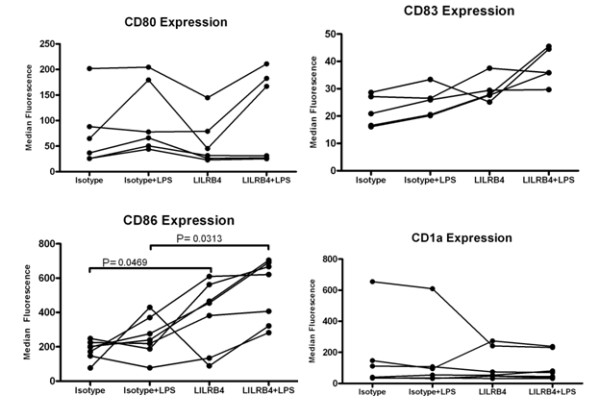
**Cell surface phenotype of LILRB4-ligated dendritic cells**. Flow cytometric analysis of cell surface markers was performed for dendritic cells cultured in the presence of an isotype control antibody (Isotype) or LILRB4-specific antibody (LILRB4) with Protein G. LPS-matured dendritic cells cultured in the presence of isotype control antibody (Isotype+LPS) or LILRB4-specific antibody (LILRB4+LPS) were also compared. Results for individual donors are indicated by joined lines. A minimum of 5 individuals were examined for each marker. Statistical analysis of isotype control versus LILRB4-ligated cells was performed using a Wilcoxon matched pairs test on Prism Software (GraphPad, USA).

Flow cytometric analysis of cell surface markers was used to determine the effects of LILRB4 ligation on the cell-surface phenotype of antigen presenting cells cultured *in vitro*. This technique has been used successfully for studies of other LILR family members [8,14,15]. Cells were cultured with a LILRB4-specific antibody (ZM3.8) and crosslinking agent (protein G), both replenished throughout culture in order to maintain receptor crosslinking. Ligation with ZM3.8 triggers LILRB4 activation and SHP-1 recruitment [5]. Ligation of LILRB4 led to an upregulation of the co-stimulatory protein CD86 on dendritic cells on both immature and LPS-matured dendritic cells (Figure 1). No significant effects on the expression of CD1a, CD80 or CD83 were observed.

**Figure 2:**
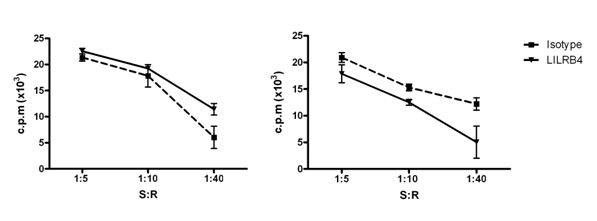
**T cell stimulatory properties of LILRB4-ligated dendritic cells**. B. Dendritic cells were cultured in the presence of isotype control or LILRB4-specific antibody with cross-linking and LPS matured as before. Naïve CD4+ T cells from an allogeneic donor were added at various stimulator:responder (S:R) ratios and cultured for 6 days. CD4+ T cell proliferation was assessed by thymidine incorporation. Triplicate wells were analysed with error bars representing the SEM. Results are shown for 2 experiments, which are representative of the 5 performed.

We also tested whether ligation of LILRB4 resulted in any cell-surface changes that might inhibit the ability of dendritic cells to stimulate naïve CD4+ T cells. Mixed leukocyte reactions (MLR) were performed using naïve CD4+ T cells isolated from allogeneic subjects. Results of five MLR experiments indicated no consistent increase or decrease in T cell proliferation in response to LILRB4 cross-linked dendritic cells (Figure 2).

### LILRB4 ligation alters the cytokine secretion profile of macrophages

**Figure 3:**
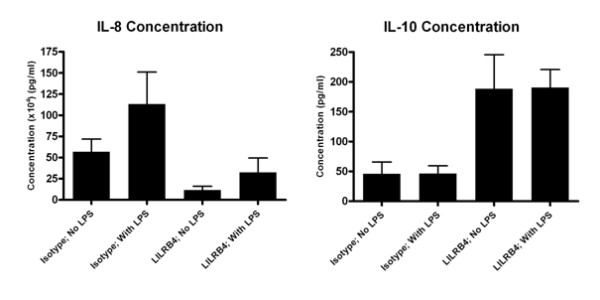
**LILRB4 ligation throughout macrophage culture reduces IL-8 but increases IL-10 production**. Cytokine levels in supernatants of *in vitro*-cultured macrophages were analysed by ELISA. Macrophages were cultured in the presence of Isotype control or LILRB4 specific antibody. Three sets of triplicate samples were analysed with error bars representing the SEM. Statistical testing by two-sided Student's *t *test did not show any significant differences between treatment groups.

We then examined the effects of LILRB4 ligation on cytokine secretion by antigen presenting cells. Supernatants were harvested from *in vitro *macrophage cultures. During an initial screen by cytokine bead array (CBA), differences in IL-8 and IL-10 production were observed. These changes were further analysed by ELISA. Although results were not statistically significant by two-sided Student's *t *test, the concentration of IL-8 in culture supernatant was seen to be decreased following LILRB4 ligation for both immature and LPS-matured cells (Figure 3). In contrast, LILRB4 cross-linking increased levels of IL-10 in the culture supernatant.

### *Salmonella typhimurium *infection upregulates expression of LILRB2 and LILRB4

**Figure 4:**
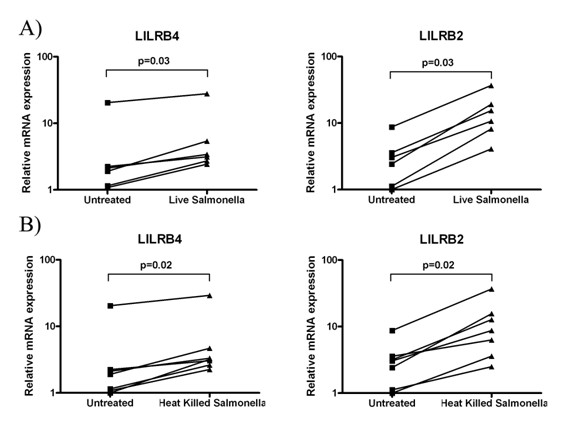
***Salmonella typhimurium *upregulates expression of *LILRB2 *and *LILRB4 *on *in vitro*-cultured macrophages**. Real-time PCR was used to assess the expression of *LILRB2 *and *LILRB4 *mRNA in monocyte-derived macrophages treated with either live or heat-killed GFP^+ ^*S. typhimurium*. *Salmonella *infection was confirmed by flow cytometic detection of GFP (data not shown). p-values were calculated using a two-tailed Wilcoxon rank test, using a 95% confidence level on Prism Software (GraphPad, USA).

**Figure 5:**
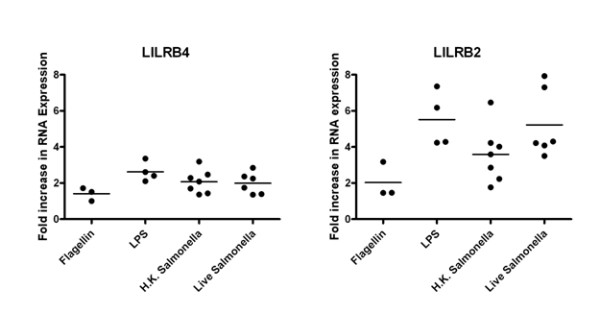
**LPS treatment upregulates expression of LILRB2 and LILRB4 at a similar level to that observed for whole bacteria**. Alterations in the mRNA expression of *LILRB4 *and *LILRB2 *in monocyte-derived macrophages following stimulation with *S. typhimurium *LPS, Flagellin, or whole *S. typhimurium *(either live or heat-killed (H.K.)).

To investigate the biology of LILRB2 and LILRB4 on antigen presenting cells in the context of infection, we infected monocyte-derived macrophages with *Salmonella typhimurium *transformed with the pFVP25.1-GFP plasmid [16]. Infection of macrophages was confirmed by GFP expression as assessed by flow cytometry. Real-time PCR analysis showed upregulation of both *LILRB2 *and *LILRB4 *in *Salmonella-*infected macrophages (Figure 4). A similar result was observed when macrophages were treated with heat-killed bacteria (Figure 5).

**Figure 6:**
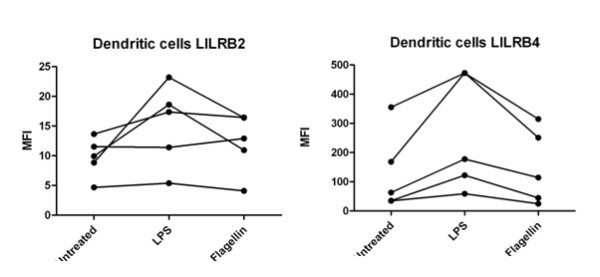
**LPS treatment upregulates LILRB2 and LILRB4 on *in vitro *cultured dendritic cells**. Flow cytometric analysis was used to study surface expression of LILRB4 and LILRB2 on *in vitro*-cultured dendritic cells following treatment with LPS or flagellin.

We then investigated whether bacterial components known to trigger Toll-like receptors (TLR) could elicit a similar response. Recognition of lipopolysaccharide (LPS) is predominantly mediated by TLR4 [17,18], and flagellin, a potent innate immune stimulator is recognised by TLR5 [19]. Treatment of *in vitro*-cultured macrophages with *Salmonella typhimurium *LPS led to an increase in *LILRB2 *and *LILRB4 *in our real-time PCR assay, similar to that observed for whole *Salmonella*. Upregulation was less pronounced for cells treated with flagellin (Figure 5). TLR stimulation was also performed on *in vitro*-cultured dendritic cells, with a general upregulation of LILRB2 and LILRB4 observed following LPS stimulation, and little or no effect for flagellin treatment (Figure 6).

## Discussion

We have shown that continuous ligation of LILRB4 during *in vitro *culture of dendritic cells led to an enhanced upregulation of CD86. This contrasts with LILRB2, where ligation results in a failure to upregulate costimulatory proteins including CD86 [8,9]. A mixed leukocyte reaction assay further confirmed that LILRB4 cross-linking did not result in any changes to the surface phenotype of antigen presenting cells that could inhibit T cell proliferation. Our results complement previous work from Suciu-Foca *et al*, which has shown that a soluble form of LILRB4 is sufficient to inhibit T cell stimulation [10,11]. Thus, although they have similar expression profiles and similar effects on T cell proliferation, LILRB2 and LILRB4 may use different modes of action to regulate T cell activity - with LILRB2 limiting the co-stimulatory potential of antigen presenting cells whilst LILRB4 acts through its T cell ligand, during T cell/antigen presenting cell contact.

Although limited effects on the surface phenotype of antigen presenting cells were observed in our study, LILRB4 cross-linking did result in some alteration of cytokine secretion profile. LILRB4 ligation led to an upregulation of IL-10 secretion by *in vitro*-cultured macrophages. This inhibitory cytokine may contribute to a feedback loop of LILR-mediated inhibition as it upregulates LILRB1, LILRB2, LILRB3 and LILRB4 on antigen presenting cells [20-22]. IL-8 is an inflammatory chemokine that attracts cells of the innate immune system [23]. That LILRB4-ligation results in increased production of IL-10, a potently anti-inflammatory factor [24], and correspondingly reduces expression of the strongly inflammatory IL-8, is consistent with its immune inhibitory nature.

The interaction between professional antigen presenting cells and antigen-specific T cells provides signals important for the efficient activation and maintenance of a T cell response but also, as evidenced by our receptor-ligation studies, provides signals important for regulation of inflammation. We propose that LILRB4 plays a dual immunoregulatory role to limit both innate and adaptive immunity; cell-cell contact would allow LILRB4 on the antigen presenting cell to deliver an inhibitory signal to T cells to regulating adaptive immunity whilst delivering an inhibitory signal to innate immune cells through decreased IL-8.

Although we saw no effect of LILRB4 cross-linked antigen presenting cells on the quantity of T cell proliferation, it is possible that there may have been qualitative differences in the T cell cultures. Further studies would be necessary to determine whether culture with LILRB4-crosslinked antigen presenting cells leads to alterations in T cell function or the ratio of, for example, regulatory or suppressor subsets.

The rapidly growing field of LILR biology has begun to address the expression and relevance of these receptors during immune dysregulation or transplant situations, but there is relatively little knowledge regarding LILR during infection. To address this, we observed the effects of *Salmonella *infection on *LILRB2 *and *LILRB4 *expression. Infection of macrophages with *Salmonella *resulted in an upregulation of LILRB2 and LILRB4. Similar effects were observed when cells were treated with heat-killed *Salmonella *or *Salmonella *LPS, which might indicate that the effects we observed during *Salmonella *infection were largely due to LPS recognition. The effects of LILR and TLR signalling on each other's expression and function is likely to be a future topic of interest in the field of innate/adaptive immune regulation; both families are predominantly expressed on the same cell types, LILR activity can inhibit TLR function [3,25], and TLR triggering can modulate LILR expression [26]. The relationship between TLR (innate immune receptors for microbes) and LILR (innate immune receptors for self) may therefore represent an immunostimulatory/immunoregulatory balance, with inhibitory LILR upregulation acting as a mechanism to prevent an over-exuberant immune response.

Antigen presenting cells show versatility in their ability to tailor an immune response specific to individual classes of pathogens. Variations in LILR expression profile, with their effects on surface phenotype and cytokine secretion, could help skew immune responses in different directions. Our experiments, and those of Cohen *et al *using *Porphyromonas gingivalis *show an upregulation of LILRB4 in response to LPS treatment [27], but stimulation with TLR9 ligands led to a downregulation of LILRB4 [26]. It is also notable that in microarray analysis for *Salmonella typhimurium *lacking a virulence factor that can promote LPS modification, LILRB4 was one of the few genes whose expression was reduced [28].

Normally, inflammation is a balanced process; the production of anti-inflammatory factors is crucial in limiting the response to the site of pathological challenge. Expression levels of LILRB2 and LILRB4 were previously shown to correlate with inhibitory activity and are enhanced in the presence of anti-inflammatory agents [4,20,29,30]. Our finding that LILRB2 and LILRB4 are upregulated in response to *Salmonella *stimulation indicates that these receptors play a role in limiting the inflammatory response during infection.

## Conclusion

Our results indicate that the inhibitory effects of LILRB4 do not result from a failure to upregulate co-stimulatory proteins. In addition to the high level expression that can render antigen presenting cells tolerogenic, there may be a role for lower level expression and activity of LILRB2 and LILRB4 in response to TLR signalling during an immune response to bacterial infection. The differing roles of LILRB2 and LILRB4 on antigen presenting cell biology and in T cell/antigen presenting cell interactions warrant future studies that should prove useful in multiple fields of immunology and infectious disease.

## Methods

### Flow Cytometry

All staining procedures were performed at 4°C. Washed cells were incubated with a selection from the following antibodies; CD1a-FITC (BD Pharmingen), CD11c-Alexafluor647 (Serotec), CD83-FITC (BD Pharmingen), CD80-PE (BD Pharmingen), CD86-Cychrome (BD Pharmingen), the unconjugated LILRB4-specific antibody ZM3.8 [5] was a generous gift from Prof. M. Colonna (Washington University, USA), LILRB2-PE (Beckman Coulter), LILRB4-PE (Beckman Coulter), Isotype control mIgG1 (Southern Biotech), Isotype control antibodies for flow cytometry (BD Pharmingen). Flow cytometric analysis was performed on a Becton Dickinson FACScan using CellQuest software.

### Culture of *in vitro*-derived macrophages and dendritic cells

Peripheral blood mononuclear cells were obtained from the National Blood Service (Cambridge, UK; St George's Hospital, UK) in the form of buffy coat and separated by density centrifugation over lymphoprep (Axis Shield, Norway). Adherent cells were cultured in RPMI1640 containing 1% autologous plasma, 50 U/ml penicillin and 50 U/ml streptomycin. Dendritic cells were cultured in 50 ng/ml of GM-CSF and IL-4 (Peprotech), macrophages in 50 ng/ml M-CSF, with cytokines and culture medium refreshed every second day. Cells were matured on day 6 of culture with 10 ng/ml LPS or as required (see below). For LILRB4 ligation, dendritic cells were cultured with 10 μg/ml of the LILRB4 specific antibody ZM3.8 or mouse IgG1 isotype control with 2 μg/ml protein G (Zymed USA) as a cross-linking agent, ZM3.8/IgG1 isotype and protein G were refreshed every second day throughout culture.

For Toll-like Receptor stimulation studies, cells cultured as above were stimulated on day 6 with 10 ng/ml *S. typhimurium *LPS (Sigma-Aldrich, UK) or 100 ng/ml *S. typhimurium *Flagellin (Invivogen, US). Cells were incubated for a further 24 hours to allow maturation.

### Cytokine analysis

An initial screen was performed using a multiplex array (Bender MedSystems, UK) for IFN-γ, IL-1β, IL-2, IL-4, IL-5, IL-6, IL-8, IL-10, IL-12p70 and TNF-α according to the manufacturer's instructions. Commercial quantitative ELISA kits (R&D systems, UK) were used to assess cytokine profiles of interest (IL-8, IL-10). The average triplicate reading for each standard, control and sample was subtracted from the average zero standard optical density reading. A standard curve was determined and used to determine the total cytokine concentration.

### Mixed leukocyte reaction

24 hours after LPS maturation, LILRB4-ligated (and isotype control-treated) dendritic cells were washed, irradiated with 50 Gy and added in various dilutions to 96 well round-bottomed microplates. Allogeneic CD4+ naïve T cells were negatively isolated using a magnetic bead negative isolation kit (Dynal, UK) and 5 × 10^4 ^cells were added to triplicate wells containing dendritic cell stimulators. Culture medium was RPMI1640 with antibiotics, L-glutamine and 5% human AB serum (Sigma-Aldrich, UK). On day 6 of culture, 1 μCi of tritiated thymidine (GE Healthcare, UK) was added and plates incubated for a further 18 hours before harvesting and analysis using a scintillation plate reader. Data points were corrected against readings for negative control wells containing only responder cells.

### Bacterial infection

*Salmonella enterica serovar Typhimurium *strain 12023 (ATCC 14028) carrying the plasmid pFVP25.1-GFP for fluorescence visualization [16] was used to infect monocyte-derived macrophages on day 6 of culture. Stationary-phase *Salmonella *were opsonised in RPMI containing 20% human autologous serum for 30 min. Cells were then washed with PBS and incubated with fresh culture medium containing gentamicin (50 μg/ml) for 1 hour. The antibiotic concentration was subsequently reduced to 5 μg/ml for the remainder of the experiment. *Salmonella *infection was confirmed by flow cytometric detection of GFP expression. This procedure was performed in parallel using *S. Typhimurium *previously heat-killed by incubation at 80°C for 10 min.

### Real time PCR analysis of LILR expression

RNA was extracted from macrophages 8 hours after TLR and *Salmonella *treatments using an RNeasy Kit (Qiagen). RNA was eluted in 50 μl of 0.2 μm filtered H2O (Sigma). All samples underwent a DNase treatment using a Turbo DNase Free kit (Ambion). cDNA synthesis was performed using Superscript III with an Oligo(dT)_20 _primer (Invitrogen) at 50°C for 1 hour, following the manufacturers standard protocol. The oligonucleotide sequences used to assess the expression levels of *LILRB2*, *LILRB4 *and the reference genes *GAPDH*, *YWHAZ *and *HPRT *are given in the Table 1. Real-time PCR reactions consisted of 10 μl of 2× QuantiTect SYBR Green solution (Qiagen), 1 ng cDNA, oligonucleotides and brought to a final volume of 20 μl with 0.2 μm filtered H2O (sigma). Real-time PCR was performed on an ABI 7500 (PE Applied Biosystems), the cycling parameters were as follows: 95°C for 15 minutes, followed by 40 cycles of 94°C for 15 s, 60°C for 30 s and finally 72°C for 33 s. The fluorescence levels of both SYBR Green and the internal passive dye ROX were acquired during the final step. Average cycle threshold (CT) values were produced using the ABI 7500 System SDS software. The relative expression level of each target transcript in differentiating DCs was assessed using qBase [31] by normalisation against all three reference genes.

**Table 1:** Real-time PCR primer mixes

**Specificity**	**Sense primer (Sequence 5'-3')**	**Conc.** **(μM)**	**Antisense primer (Sequence 5'-3')**	**Conc.** **(μM)**
*LILRB2*	NK1009 CAGTCATGGCGGCAGTTC	0.9	NK495 GCAGGTAGGGGTCGGAGT	0.9
*LILRB4*	NK620 CTGTGTCAGTCACGGAGCC	0.9	NK621 GCAGGTAGTGGGAGAAGCC	0.9
*YWHAZ*	C20 ACTTTTGGTACATTGTGGCTTCAA^c^	0.6	C21 CCGCCAGGACAAACCAGTAT^c^	0.6
*HPRT*	C30 GTAGCCCTCTGTGTGCTCAA	0.6	C31 TCACTATTTCTATTCAGTGCTTTGATG	0.6
*GAPDH*	C28 ACACCCACTCCTCCACCTTT	0.6	C29 TGACAAAGTGGTCGTTGAGG	0.6

^a ^Sequence originally published in Vandesompele *et. al*. [32]

## Abbreviations

ILT: Immunoglobulin like transcript; LILR: Leukocyte Ig-like receptor; LPS: lipopolysaccharide; MLR: mixed leukocyte reaction; TLR: toll like receptor.

## Competing interests

The authors declare that they have no competing interests.

## Authors' contributions

DPB, DCJ, KJA and RAB carried out antigen presenting cell culture and flow cytometry. DCJ performed PCR studies. DPB performed cytokine studies and MLR. NL performed Salmonella infections. JT and RLA planned studies and RLA prepared the manuscript. All authors read and approved the final manuscript.
